# Signatures of neutral evolution in exponentially growing tumors: A theoretical perspective

**DOI:** 10.1371/journal.pcbi.1008701

**Published:** 2021-02-11

**Authors:** Hwai-Ray Tung, Rick Durrett

**Affiliations:** Department of Mathematics, Duke University, Durham, North Carolina, United States of America; University of California Irvine, UNITED STATES

## Abstract

Recent work of Sottoriva, Graham, and collaborators have led to the controversial claim that exponentially growing tumors have a site frequency spectrum that follows the 1/*f* law consistent with neutral evolution. This conclusion has been criticized based on data quality issues, statistical considerations, and simulation results. Here, we use rigorous mathematical arguments to investigate the site frequency spectrum in the two-type model of clonal evolution. If the fitnesses of the two types are λ_0_ < λ_1_, then the site frequency spectrum is *c*/*f*^*α*^ where *α* = λ_0_/λ_1_. This is due to the advantageous mutations that produce the founders of the type 1 population. Mutations within the growing type 0 and type 1 populations follow the 1/*f* law. Our results show that, in contrast to published criticisms, neutral evolution in an exponentially growing tumor can be distinguished from the two-type model using the site frequency spectrum.

## Author summary

For many years, the dominant paradigm was that cancers evolve by a succession of selective sweeps in which new fitter mutants take over the system. About five years ago, Sottoriva et al introduced the Big Bang model of cancer initiation, which postulated that all the mutations needed were present when the tumor started growing. A consequence of this viewpoint is that mutations in the growing tumor are neutral. Many researchers have objected to this conclusion for a wide variety of reasons. Here, we use mathematical analysis to show that with enough sequence data the site frequency spectrum can be used to distinguish neutral evolution from the two-phase model of clonal evolution. This conclusion differs from previously published simulation results.

## Introduction

Following up on the introduction of the Big Bang model by Sottoriva et al [1], Sottoriva and Graham [2] described what they called “a pan-cancer signature of neutral tumor evolution:” the number of mutations with frequency ≥ *f* will have the form *c*/*f*. The derivation of this result is remarkably simple and is given in Methods. In 2016, Williams et al. [3] found that 323 of 904 samples from 14 cancer types showed excellent straight line fits when the cumulative number of mutations of frequency ≥ *f* is plotted versus 1/*f*. See Fig 2B in [3]. This paper has been cited 200 times, but among these works, there are a number of papers criticizing the result. See [4–6]. The December 2018 issue of Nature Genetics contains three letters raising objections to the conclusion [7–9]. Four common criticisms are

1. Inferring the allele frequency *f* requires accurate estimates of local copy number and ploidy. In addition, Wu et al [5] point out that local samples may not be indicative of overall frequencies.
2. Failure to reject the null model is not the same as proving it is true. To quote McDonald, Chakrabarti, and Michor [8] “The fact that a model of neutral evolution leads to a linear relationship between *M*(*f*) (the number of mutations with frequency ≥ *f*) and 1/*f* does not imply … the presence of neutral evolution.”
3. Tarabichi et al [7] applied methods that look at the *dN*/*dS* ratio, which compares the number of nonsynonymous and synonymous mutations, to look for signs of selection. They claim to have found significant signs of selection in tumors that were classified as neutral. However when the analysis was repeated on publicly available pancreatic cancer data, Graham, Sottoriva et al found no values significantly different from 1.
4. Tarabichi et al [7] say “the deterministic models of tumor growth described by Williams et al [3] rely on strong biological assumptions. Using simple branching process to simulate neutral and nonneutral growth, they show that *R*^2^ > 0.98 is neither necessary nor sufficient for neutral evolution.”

To try to shed some light on the controversy, we will do a mathematically rigorous computation of the site frequency spectrum produced by the two-type model of clonal evolution. We will describe the model in Results. The two-type model and its *m*-type generalization have been extensively studied. See [10] for results and references. This model is relevant to the discussion of [3] because it appears in the criticisms of McDonald, Chakrabarti, and Michor [8] and Bozic, Patterson, and Waclaw [6]. Before we describe the math, we want to make it clear that that this work only discusses the theoretical aspects of cancer genomics and is not concerned with practical problems in making inferences on cancer genomic data, which of course could hide some of the theoretical effects due to errors, bias, sampling, and other issues discussed in the criticisms listed above.

## Results

### A two-type model

McDonald, Chakrabarti, and Michor [8] consider two alternative evolutionary models in order to argue that other underlying models can produce a linear relationship between 1/*f* and the cumulative number of mutations with frequency ≥ *f*. Their second model is an infinite alleles branching process model previously studied by McDonald and Kimmel [11]. We will ignore this model, since in studying DNA sequence data the appropriate mutation scheme is the infinite sites model.

In their first model, clonal expansion begins with a single cell of the original tumor-initiating type (type 0). To make it easier to connect with previous mathematical work, we will describe their model using the notation used in [10] and [12]. We suppose that type 0 individuals give birth at rate *a*_0_ and die at rate *b*_0_, so the exponential growth rate is λ_0_ = *a*_0_ − *b*_0_. For simplicity, we will suppose that neutral mutations accumulate during the individual’s life time at rate *ν*, instead of only at birth.

Type 0 individuals mutate to type 1 at rate *u*_1_. Type 1 individuals give birth at rate *a*_1_ and die at rate *b*_1_. Their exponential growth rate is λ_1_ = *a*_1_ − *b*_1_ where λ_1_ > λ_0_. In [8], different type 1 families have different increases in their growth rates that follow a normal distribution. In this section, we will assume all type 1 mutations have the same growth rate. Later, we will consider the implications of random fitness changes for the behavior of the model.

The reader will see many complicated formulas in this paper, so it will be useful to have a concrete set of parameters to plug into these formulas. Borrowing an example from [10], we will set

$$\begin{array}{c} a_{0}=a_{1}=1,\;\lambda_{0}=0.02,\;\lambda_{1}=.04,\;u_{1}=10^{-6},\;\nu =10^{-4}. \end{array}$$ (1)

We do not pretend that these parameters apply to any specific cancer, but for a mental picture, you can imagine that type 0s are colon cancer cells in which both copies of APC have been knocked out, while type 1 cells in addition have a KRAS mutation.

#### Limit theorems

As in [8], we will, for simplicity, restrict our attention to two types. The type 0’s are a simple branching process, so well-known results show that

$$\begin{array}{c} e^{-\lambda_{0}t}Z_{0}\left(t\right)\to W_{0}, \end{array}$$ (2)

where *W*_0_ = 0 with probability *b*_0_/*a*_0_ and has a rate λ_0_/*a*_0_ exponential distribution with probability λ_0_/*a*_0_.

The study of the second wave is simpler if we suppose that $Z_{0}^{*}\left(t\right)=V_{0}e^{\lambda_{0}t}$ for all *t* ∈ (−∞, ∞), where *V*_0_ has the same distribution as (*W*_0_|*W*_0_ > 0), that is exponential with rate λ_0_/*a*_0_. Mutations from type 0 to 1 occur at rate *u*_1_. Let *σ*_1_ be the time of the first successful type 1 mutation, i.e., one whose branching process does not die out. Durrett and Moseley [13] showed, see (29) in [10], that *σ*_1_ has median

$$\begin{array}{c} s_{1/2}^{1}=\frac{1}{\lambda_{0}}\log(\frac{\lambda_{0}^{2}a_{1}}{a_{0}u_{1}\lambda_{1}}). \end{array}$$ (3)

In the concrete example, $s_{1/2}^{1}=460.51$. In colon cancer where cells divide every four days, $s_{1/2}^{1}$ is 1842 days or a little more than 5 years.

Durrett and Moseley were the first to rigorously prove results about the asymptotic behavior of the size of the type 1 population $Z_{1}^{*}\left(t\right)$, see Section 9 of [10]. Durrett [12] noticed that the constants are simpler if we use a different normalization. Here we are assuming *a*_0_ = *a*_1_ = 1 to simplify the constants.

**Theorem 1** *As t* → ∞, $e^{-\lambda_{1}\left(t-s_{1/2}^{1}\right)}Z_{1}^{*}\left(t\right)\to {\bar{V}}_{1}$ *where* ${\bar{V}}_{1}=e^{\lambda_{1}s_{1/2}^{1}}V_{1}$ *is the sum of the points in a Poisson process with mean measure*

$$\begin{array}{c} \bar{\rho }\left(x,\infty \right)=\rho \left(e^{-\lambda_{1}s_{1/2}^{1}}x,\infty \right). \end{array}$$

Using Eq (3), and doing some algebra

$$\begin{array}{c} \bar{\rho }\left(x,\infty \right)=\alpha \lambda_{0}\lambda_{1}^{-\alpha }\Gamma \left(\alpha \right)V_{0}x^{-\alpha }. \end{array}$$

In our concrete example, $\bar{\rho }\left(x,\infty \right)=0.1772V_{0}x^{-1/2}.$ Note that due to shifting time by $s_{1/2}^{1}$, the measure $\bar{\rho }$ does not depend on the mutation rate.

#### Site frequency spectrum

There are three classes of mutations in the two-phase model

- type 0: Neutral mutations that occur to type 0 individuals.
- type 1A: Advantageous mutations that turn type 0 individuals into type 1.
- type 1: Neutral mutations that occur to type 1 individuals.

By the argument in Methods, the type 0 mutations will have a 1/*f* site frequency. The argument can also be used to prove the next result so the details are hidden away in Methods.

**Theorem 2** *The number of type 1 mutations with frequency* ≥ *f with in the type 1 population will be asymptotically ν*/(λ_1_ *f*).

The points in the Poisson process in Theorem 1 indicate the contributions of the various type one families to the limit ${\bar{V}}_{1}$, so if we let *x*_1_ > *x*_2_ > *x*_3_… be the points, then the *j*th largest family makes up a fraction $x_{j}/{\bar{V}}_{1}$ of the population. Intuitively, this implies that the number of type 1A mutations with frequency ≥ *f* will be asymptotically *Cf*^−*α*^ where *α* = λ_0_/λ_1_. However, the fact that the sum of the points in the Poisson process is random makes this difficult to study. Fortunately for us, the work has already been done in 1997 by Pitman and Yor [14], who proved that the points in the Poisson process divided by their sum follow the Poisson-Dirichlet distribution *PD*(*α*, 0). See the remark after Theorem 5 in [12]. This gives us that when 0 < *α* < 1 the site frequency spectrum of 1A mutations is:

$$\begin{array}{c} SFS_{1A}\left(f\right)=\frac{\text{sin}(\pi \alpha )}{\pi \alpha }{\left(\frac{1}{f}-1\right)}^{\alpha }. \end{array}$$ (4)

When *α* = 1/2, the constant is 2/*π* = 0.6366.

Including type 0 passenger mutations in type 1*A* families does not significantly change the *f*^−*α*^ shape in (4). This is because all important 1A mutations happen soon after the first mutation, which implies that all important 1A mutations have roughly the same number of passengers. See Methods.

**Fig 1:**
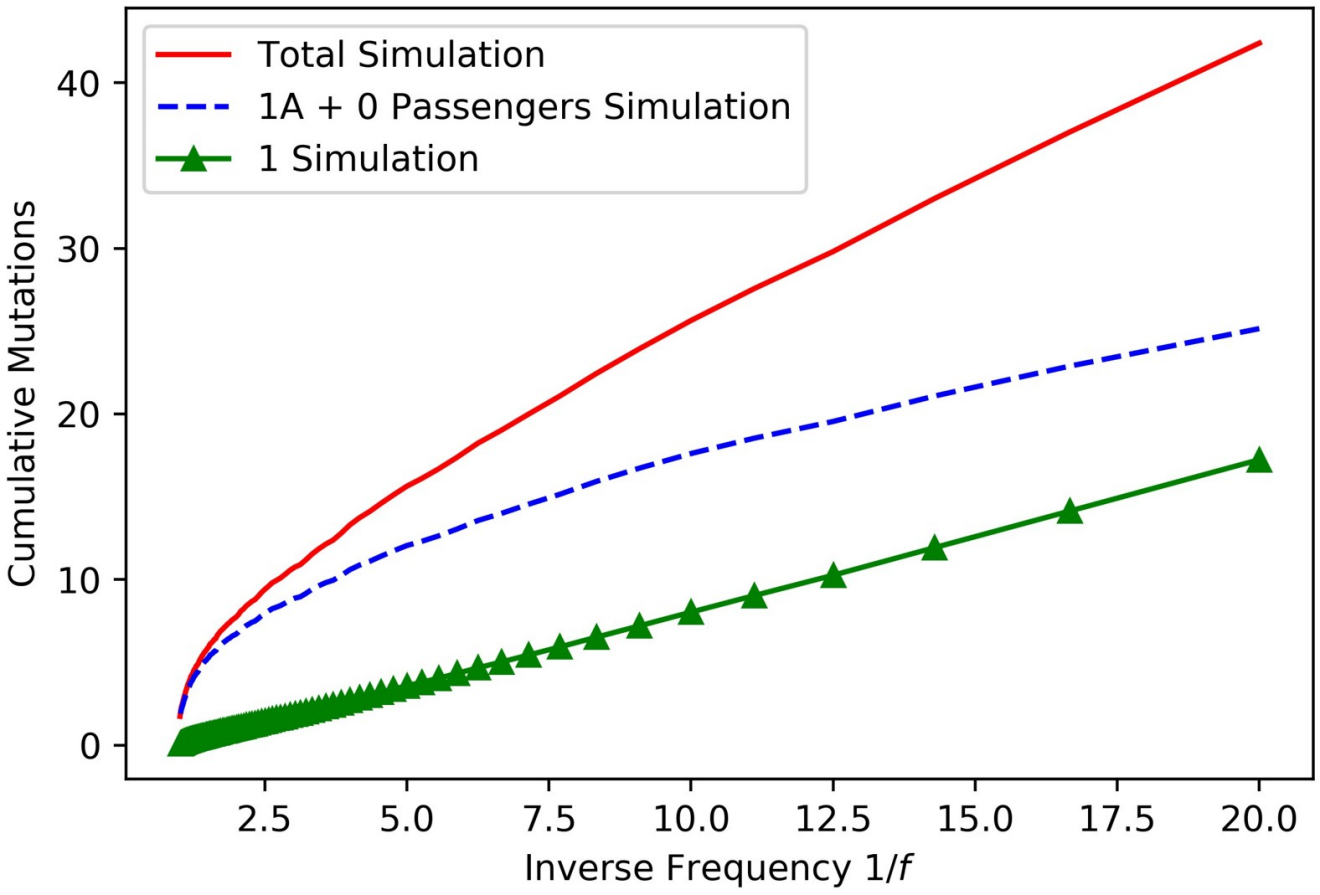
Site frequency spectrum in the type 1 population. The figure shows the contribution of the different mutation types to the site frequency spectrum. The simulation was performed with parameters *ν* = 0.02, *u*_1_ = 2 × 10^−4^, λ_0_ = 0.02, λ_1_ = 0.04 and *a*_0_ = *a*_1_ = 1 and is the average site frequency spectrum of 1000 runs. We simulated the 1*A* families and type 0 passenger mutations on their founders. Then, we obtained type 1 mutations for each 1*A* family by applying (8) in Methods. We only consider mutations present in the type 1 population because, as *t* → ∞, the proportion of the population that is type 0 cells approaches 0. As suggested from Theorem 2, the type 1 site frequency spectrum is linear when plotted against 1/*f*. The 1*A* + 0 line looks similar to a power law, as suggested by (4).

**Fig 2:**
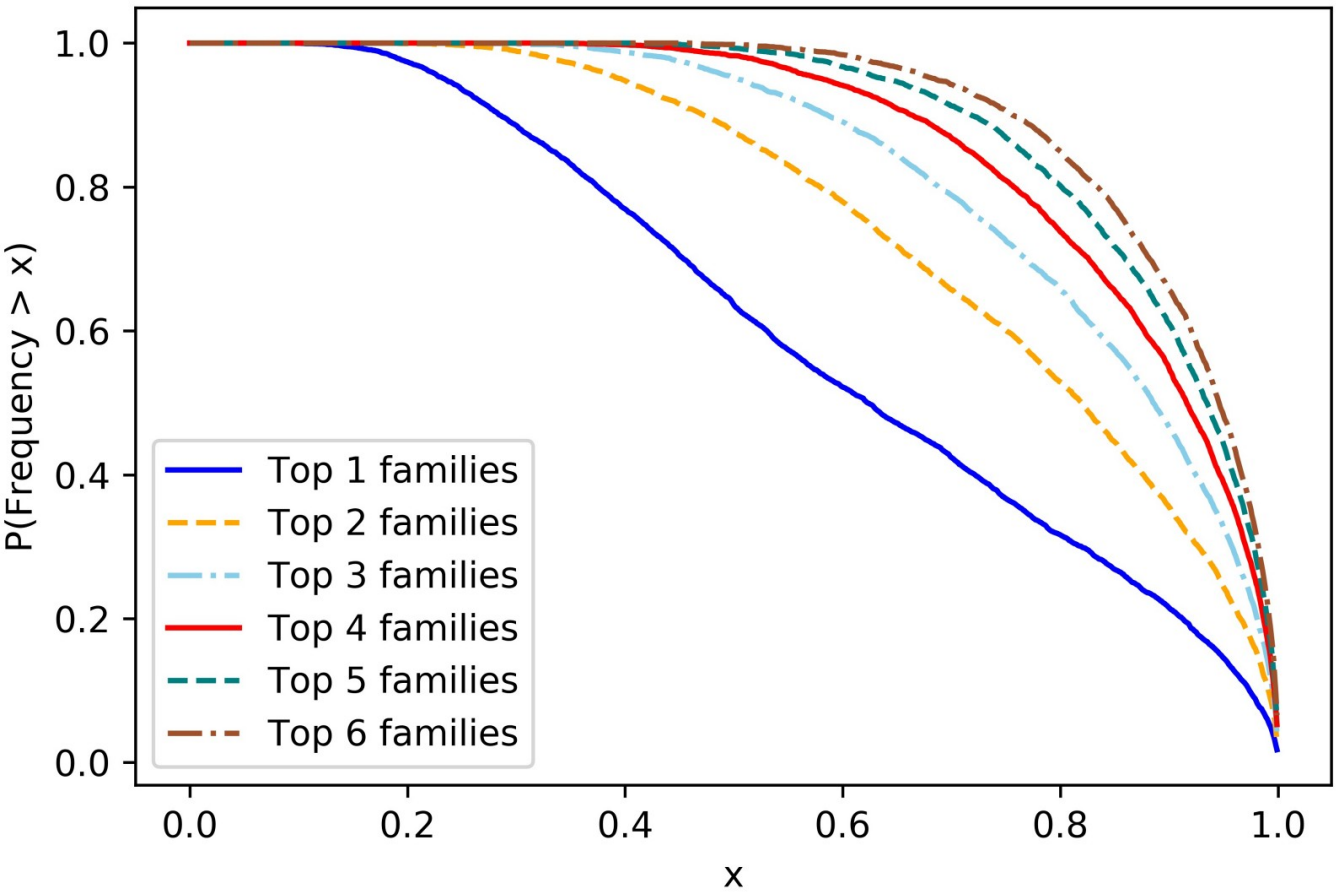
Distribution of 1A family sizes in the type 1 population. To better understand the distribution of 1*A* family sizes, we used the Poisson-Dirichlet(*α*, 0) distribution to generate the six largest families. The plot gives the probability that the number of individuals in the top *i* families are greater than a fraction *x* of the total type 1 population.

To illustrate the results proved above, we turn to simulations seen in Figs 1 and 2.

### Random fitness increases

McDonald, Chakrabarti, and Michor [8] considered the case in which type 1 individuals have growth rates that are normal with mean *m* and standard deviation *d*. Early work on models with random fitness increases in the two-type model led to very unusual behavior in the limit *t* → ∞, see [15]. Results in that paper show

- If the fitness distribution was bounded then, as *t* → ∞, individuals with fitnesses that were close to the upper limit dominated the population.
- If the distribution was unbounded, then the population could grow faster than exponential.

In this section, we will modify our example from Fig 1 so that type 1 individuals have growth rates drawn from the normal distribution with mean *m* = 0.04 and standard deviation *d* = 0.005. We will see that in contrast to the limiting results just mentioned, random fitnesses do not substantially change the behavior.

**Fig 3:**
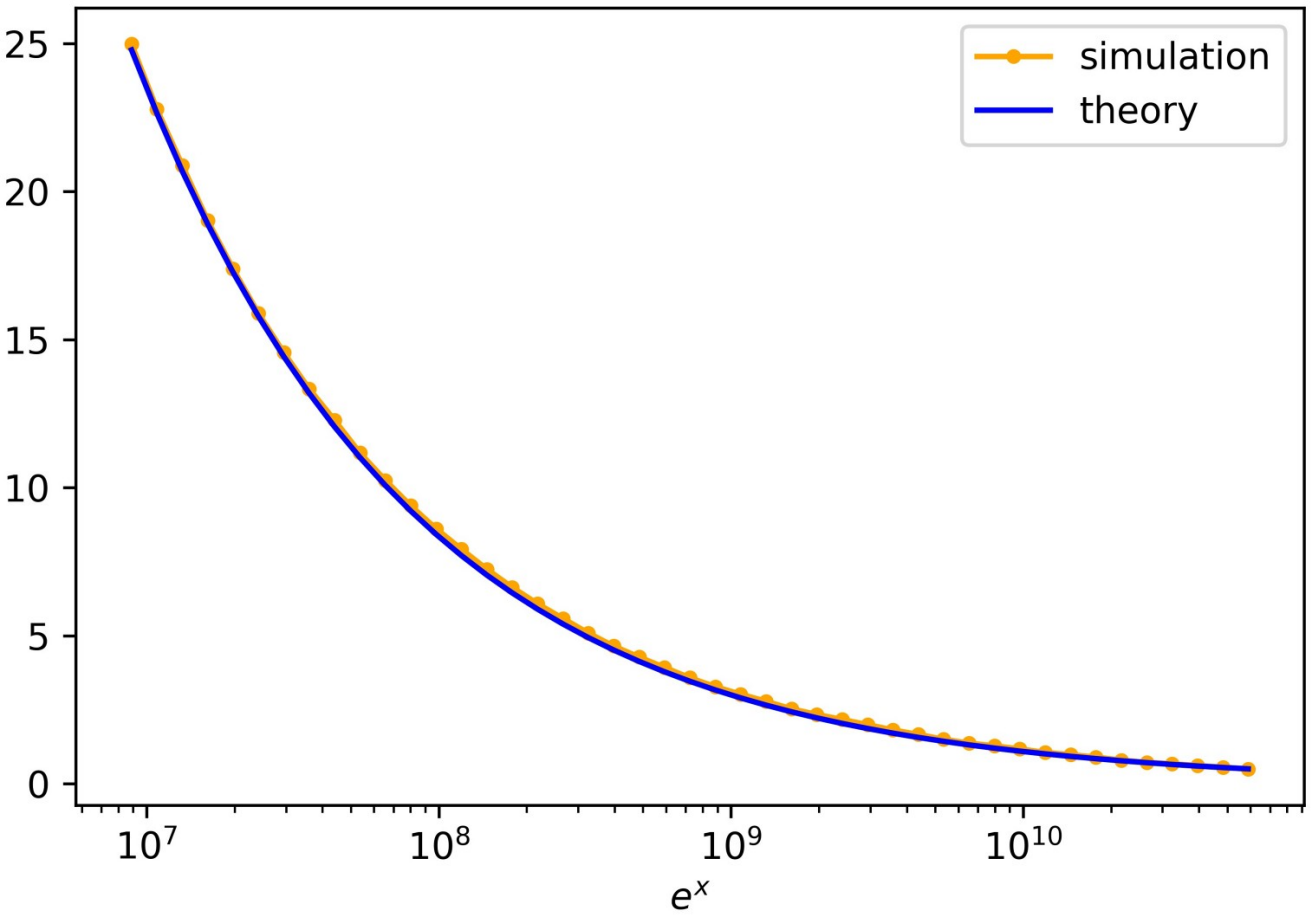
Size of 1A families with random fitness. The graph indicates the expected number of 1*A* families with λ_1,*i*_(1000 − *s*_*i*_) > *x*. The parameters are almost the same as in (1); rather than a single λ_1_ for all type 1 families, we have a different λ_1,*i*_ for each type 1A family. Each λ_1,*i*_ is normally distributed with mean 0.04 and standard deviation 0.005. 500 runs were done up until time *t* = 1000. The graph shows that on average there is one family with *e*^*x*^ > 10^10^. If the λ_1,*i*_ of the largest family is within 2 standard deviations, then multiplying *e*^*x*^ by 1/λ_1,*i*_ implies a family of magnitude around 2 × 10^11^ or greater.

To find the distribution of the growth rates of the mutations with the largest family sizes, we note that a mutant that occurs at time *s*_*i*_ and has growth rate λ_1,*i*_ will grow to size *W*_1_exp(λ_1,*i*_(1000 − *s*_*i*_)) at time 1000. The number of *i* that are successful and have λ_1,*i*_(1000 − *s*_*i*_) > *x* is Poisson with mean given by the following integral

$$\begin{array}{cc} & 10^{-6}\int_{0}^{1000}50e^{0.02s}\int_{x/(1000-s)}^{\infty }\lambda \phi \left(\lambda \right)\;d\lambda ds\\ & =10^{-6}\int_{0}^{1000}50e^{0.02s}[0.04(1-\Phi (\frac{x}{1000-s}))+0.005^{2}\phi (\frac{x}{1000-s})]ds. \end{array}$$ (5)

where *ϕ* and Φ are the density function and distribution function, of a normal distribution with mean *m* = 0.04 and standard deviation *d* = 0.005. The equality follows from substituting *u* = (λ − 0.04)^2^ for the inner integral. Fig 3 graphs (5).

**Fig 4:**
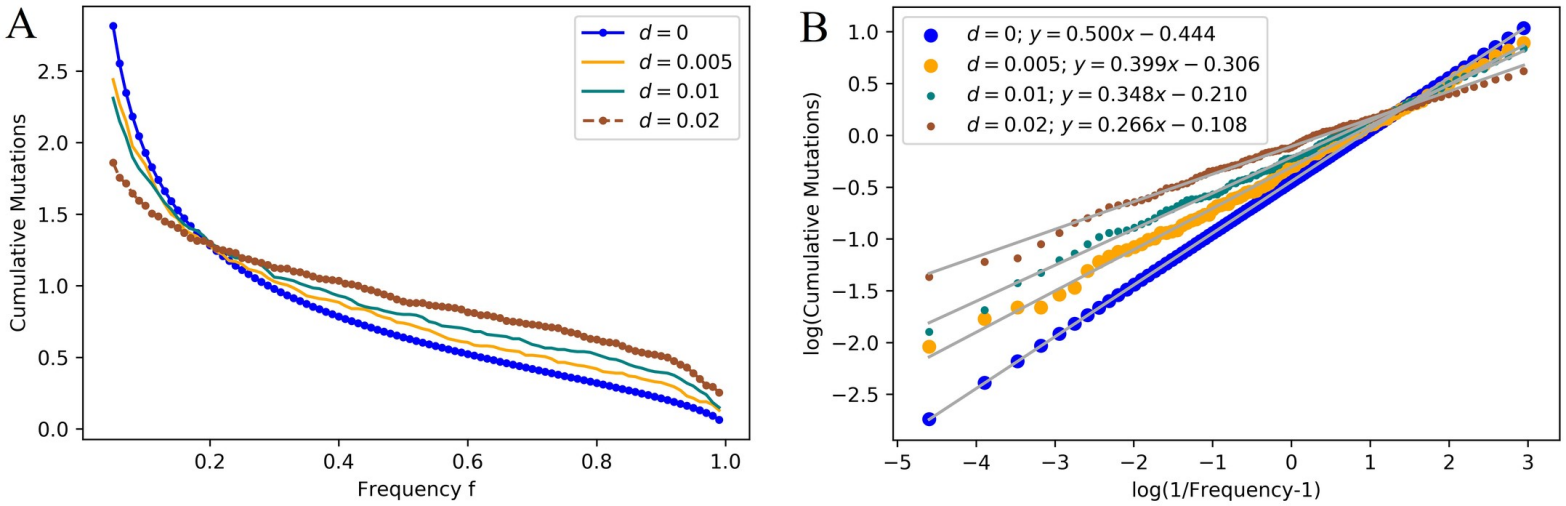
Site frequency spectrum with random fitnesses. (A) shows the site frequency spectrum for multiple values of *d*. The other parameters are the same as in Fig 3. As the contribution from neutral mutations is negligible, we will only show the contribution from 1*A* families. The line for constant, i.e., *d* = 0, is plotted from theory; the others are plotted from simulations with 200 runs. As *d* increases, the expected size of the frequency of the largest mutation increases. Also, fewer mutations reach above the 0.05 frequency threshold. (B) displays the same data on a log-log plot. The slopes *β* of the linear fits indicate that the site frequency spectrum takes the form *C*/*f*^*β*^, with *β* decreasing as *d* increases.

The random fitnesses cause the relative sizes of the contributions of mutations to the final population to change, but as Fig 4 shows, the site frequency still has the form *C*/*f*^*β*^, where *β* ≤ *α* and achieves equality in the case of non-random changes, i.e. *d* = 0.

The authors of [8] claim that the site frequency spectrum in the two-type model is 1/*f*. However, their simulation methods take the very crude approach of considering the binary split process until 1,000 or 1,000,000 cells are produced. This corresponds to 10 and 20 generations respectively. To make it possible for something to happen in this short amount of time the mutation rate for advantageous mutations is set to be 0.1 in the 1000 cell scenario, and to 0.03 when there are 1,000,000 cells. At birth, each cell acquires a Poisson mean 100 number of mutations. In contrast our simulations run for approximately 1000 generations, leading to populations of order 10^9^ cells, and neutral mutations occur slowly, leading to genealogical relationships that are more like those found in growing cancer tumors.

### Subclonal mutation frequencies

Bozic, Paterson, and Waclaw [6] argue that “the fact that no subclonal driver is present at intermediate frequencies cannot be taken as proof of neutral or *effectively neutral* evolution. It can be a consequence of population dynamics which create only a short window during which the driver mutation can be detected but not fixed in the population.” In this section we will describe their results and give a simple analytic derivation.

To argue for this viewpoint, they use the two-type model but with different notation

$$\begin{array}{llllllll} \text{here} & a_{0} & b_{0} & \lambda_{0} & a_{1} & b_{1} & \lambda_{1} & u_{1}\\ \left[6\right] & b & d & r & b_{1} & d_{1} & r_{1} & u \end{array}$$

**Fig 5:**
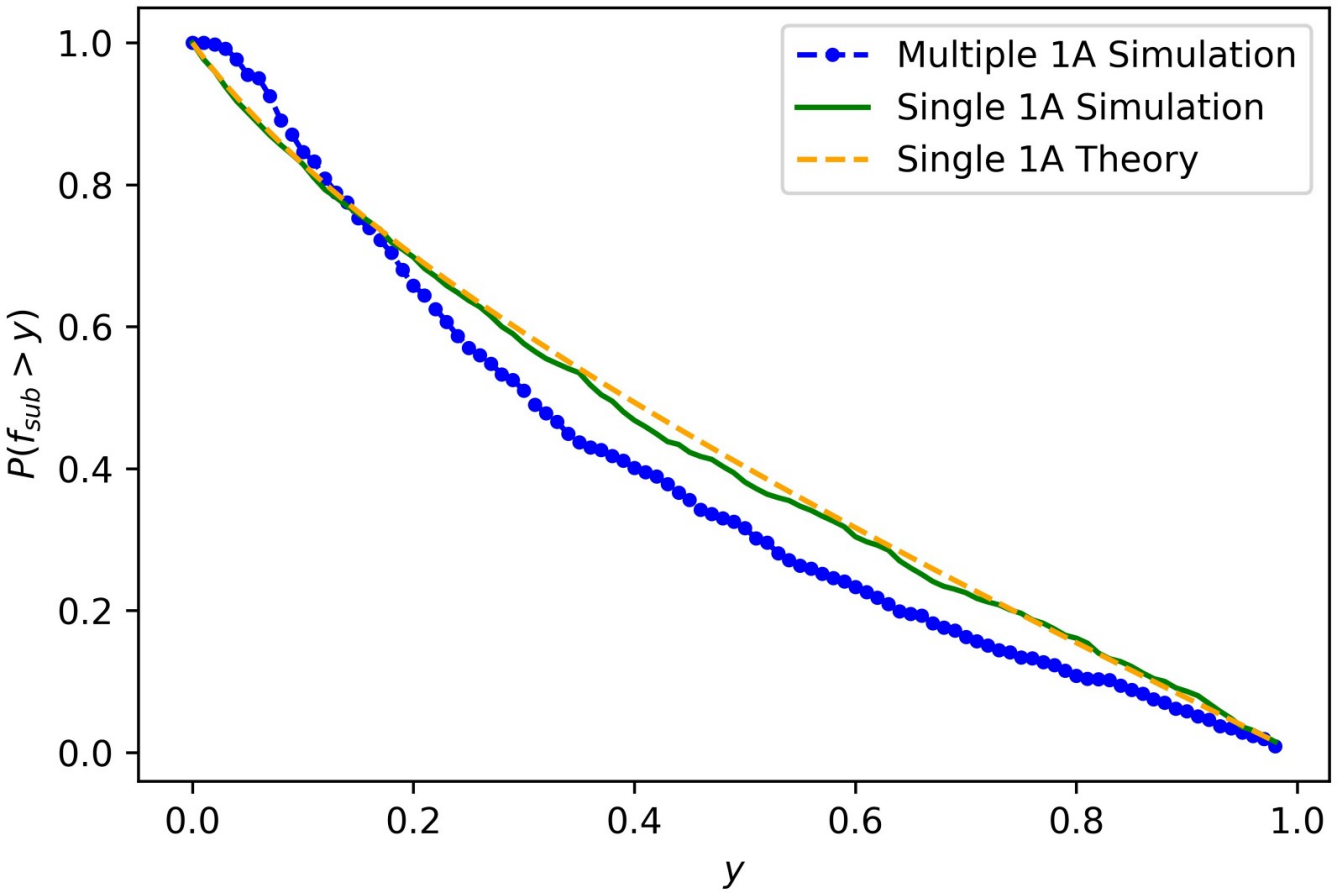
Driver frequencies. This graph gives the probability of having a driver with frequency greater than *y* once the tumor reaches size 10^9^. The parameters used are *a*_0_ = *a*_1_ = 1, λ_0_ = 0.02, λ_1_ = .035 and *u*_1_ = 10^−5^ and the data was generated from 1000 runs. Single 1A refers to approach taken by Bozic et al. where there is only 1 selective mutation. Multiple 1A is our approach. The theory curve comes using a Riemann sum with interval size 500 to evaluate the integral in Eq (6).

In addition they define *c* = *r*_1_/*r* > 1, and *g* = *c* − 1. They assume that the mutation to type 1 occurs at time 0 and run the process until the time *t* at which the total population size is *M*. Let *X*_0_ be the population of type 0’s when the mutation occurs. Since *X*_0_ is large, *X*_*t*_ ≈ *X*_0_ *e*^*rt*^. The type 1 population at time *t* is *Y*_*t*_ ≈ *W*_1_ *e*^*rct*^, where *W*_1_ is an exponentially distributed random variable with rate *cr*/*b*_1_. Note that as in Bozic et al [16] the possibility of subsequent driver mutations is ignored. As Fig 5 shows, that change does not lead to a substantial error.

Writing *f*_*sub*_ = *Y*_*t*_/(*X*_*t*_ + *Y*_*t*_) they prove that when the total tumor size is *M* = *X*_*t*_ + *Y*_*t*_ the subclonal mutation frequency has

$$\begin{array}{c} P\left(f_{sub}\leq y\right)=\int_{0}^{M}\left(uc/b_{1}\right)\exp\left(-ucx_{0}/b_{1}\right)[1-\exp(-\frac{cr}{b_{1}}\frac{y}{{\left(1-y\right)}^{c}}x_{0}^{c}M^{1-c})]dx_{0}, \end{array}$$ (6)

which is (1) in [6]. From this they can compute the probability of a subclonal driver being detectable, that is, *P*(0.2 ≤ *f*_*sub*_ ≤ 0.8).

To see what this complicated formula implies, the authors turn to simulation. The mutation rate to produce an additional driver is *u* = 10^−5^. Their Fig 2A shows a moderately growing tumor *b* = 0.14, *r* = 0.01, 2B a fast growing tumor *b* = 0.25, *r* = 0.07, and 2C a slowly growing tumor *b* = 0.33, *r* = 0.0013. For moderate values of selection, e.g. *g* = 30%, the probability that a driver mutation is in the detectable range [0.2, 0.8] is < 15% for population sizes up to *M* = 10^9^ cells and remain below 1/3 for *M* ≤ 10^11^. For other cases considered there (*g* = 70% and 100%) the chance of detecting the subclonal driver is always < 60% and for a broad range of sizes is less than 30%. Panels d,e,f in their Fig 2 show the frequency of a subclonal driver in the case of moderate growth when the size *M*_*d*_ = 10^7^, *M*_*e*_ = 5 ⋅ 10^10^ and *M*_*f*_ = 2 ⋅ 10^8^. In the three cases the frequency is near 0, near 1, and almost uniformly distributed on [0, 1].

Rather than study the tumor when it reaches a fixed size, we will derive results at a fixed time by using Theorem 1. Recall that we have set $Z_{0}^{*}\left(t\right)=V_{0}e^{\lambda_{0}t}$ and have shown

$$\begin{array}{c} e^{-\lambda_{1}\left(t-s_{1/2}^{1}\right)}Z_{1}^{*}\left(t\right)\to {\bar{V}}_{1}. \end{array}$$

Combining the last two results, we see that

$$\begin{array}{c} r\left(t\right)=\frac{Z_{1}^{*}\left(t\right)}{Z_{0}^{*}\left(t\right)}\approx e^{-\lambda_{0}t}e^{\lambda_{1}\left(t-s_{1/2}^{1}\right)}{\bar{V}}_{1}/V_{0}. \end{array}$$

Inserting the values of the λ_*i*_

$$\begin{array}{c} \frac{r(t+s)}{r(t)}=e^{(\lambda_{1}-\lambda_{0})s}=e^{0.015s} \end{array}$$

so $Z_{1}^{*}\left(t\right)/Z_{0}^{*}\left(t\right)$ goes from 0.2/0.8 = 1/4 to 0.8/0.2 = 4 in time ln(16)/0.015 = 184, confirming that the window in which competing subclones coexist is short.

## Discussion

Work of Sottoriva and Graham [2] and their co-authors [3] has shown that in many cases an exponentially growing tumor has a 1/*f* site frequency spectrum. This result has a simple derivation but the claim has drawn a large amount of criticism. Many of these concern the quality of the data used. Here, we have performed a mathematical analysis to show that given enough sequence data the site frequency spectrum can be used to distinguish neutral evolution from one specific type of selection. This analysis provides a useful complement to studies based solely on simulation.

We have studied the two-type model of cancer evolution in which the exponentially growing population of type 0 cells can mutate to a fitter type 1, and all cells can experience neutral mutations. In this model there are three types of mutations that we call 0, 1*A*, and 1. Type 0 mutations are neutral, occur to type 0 individuals, and have a 1/*f* site frequency spectrum. Type 1 mutations are neutral, occur to type 1 individuals, and again have a 1/*f* site frequency spectrum. Type 1A mutations are selective, occur to type 0 individuals, and result in type 1 individuals. When the two types have growth rates λ_0_ < λ_1_, where *α* = λ_0_/λ_1_, then the site frequency spectrum has the shape 1/*f*^*α*^ due to 1A mutations and the type 0 neutral mutations present in the founders of the type 1 population. These mutation types are more numerous than the others.

McDonald, Chakrabarti, and Michor [8] have used the two-type model to suggest that models with selection can have a 1/*f* site frequency spectrum. Our results show this is not true when type 1 mutations all have the same fitness increase. Their model has random increases in fitness, but we also show that this feature does not significantly change the qualitative features of the site frequency spectrum.

Bozic, Paterson, and Waclaw [6] study the two-type model and show that it is difficult to capture a subclonal driver mutation at intermediate frequency. Their model allows only one type 1A mutation. Using our simple analytical results and computer simulations, we confirm that this prediction holds in the two type model without that restriction.

## Methods

### Simple derivations of the 1/*f* spectrum

Sottoriva and Graham say in their original paper [2] that “the power law signature is common to multiple tumor types and is a consequence of the effectively-neutral evolutionary dynamics that underpin the evolution of a large proportion of cancers.” To explain the source of the 1/*f* curve in an exponentially growing tumor, we give the derivation of the 1/*f* frequency distribution from [3]. They assumed that cells divide at rate λ and use *N*(*t*) to be the number of cells at time *t*. If we assume that the mutation rate is *μ* (which we assume takes into account their ploidy parameter *π*), then the expected number of new mutations before time *t*, *M*(*t*), satisfies

$$\begin{array}{c} \frac{dM}{dt}=\mu \lambda N\left(t\right). \end{array}$$

Solving gives

$$\begin{array}{c} M\left(t\right)=\mu \lambda \int_{0}^{t}N\left(s\right)\;ds. \end{array}$$

Since *N*(*s*) = *e*^*λs*^ (we have set *β* in [3] to be 1 for simplicity), we observe that a mutation that occurs at time *s* will have frequency *e*^−*λs*^ in the population. Evaluating the integral in the previous formula, we have

$$\begin{array}{c} M\left(t\right)=\mu \left(e^{\lambda t}-1\right). \end{array}$$

Ignoring the −1, if we set *t*_*f*_ = −(1/λ)log *f* to make *N*(*t*_*f*_) = 1/*f* so that mutations before time *t*_*f*_ will have frequency ≥ *f*, then

**Theorem 3** *The number of mutations with frequency* ≥ *f is*

$$\begin{array}{c} M(t_{f})=\mu /f. \end{array}$$ (7)

Note that in this derivation, mutations occur only at birth. If we instead let mutations happen continuously throughout a cell’s lifetime and call the mutation rate *ν*, then Durrett [12] has shown

$$\begin{array}{c} M\left(t_{f}\right)=\frac{\nu }{\lambda f}. \end{array}$$ (8)

From the derivation given above, we see that the 1/*f* site frequency spectrum comes from the fact that mutations occur at a rate proportional to the size of the population and the fact that the population is growing exponentially fast.

### Proof of Theorem 2

*Proof.* We follow the derivation of Theorem 3. If we let $N\left(s\right)=Z_{1}^{*}\left(s\right)$, then the number of type 1 mutations by time *t* satisfies

$$\begin{array}{c} \begin{array}{cc} M_{1}\left(t\right) & =\nu \int_{s_{1/2}^{1}}^{t}N\left(s\right)\;ds\approx \nu {\bar{V}}_{1}\int_{s_{1/2}^{1}}^{t}\exp\left(\lambda_{1}\left(s-s_{1/2}^{1}\right)\right)\;ds\\ & \approx \nu {\bar{V}}_{1}\exp\left(\lambda_{1}\left(t-s_{1/2}^{1}\right)\right)/\lambda_{1} \end{array} \end{array}$$

where we have again dropped the −1 that comes from the lower limit. A mutation that occurs at a time $t\leq t_{f}=s_{1/2}^{1}-\left(1/\lambda_{1}\right)\log\left(f{\bar{V}}_{1}\right)$, when there are

$$\begin{array}{c} \leq N\left(t_{f}\right)\approx {\bar{V}}_{1}\exp\left(\lambda_{1}\left(t_{f}-s_{1/2}^{1}\right)\right)={\bar{V}}_{1}\exp\left(-\log\left(f{\bar{V}}_{1}\right)\right)=1/f \end{array}$$

individuals, will occur in a fraction of ≥ *f* of the population, so computing *M*(*t*_*f*_) gives the desired result.

### Passengers do not change the shape of the SFS

To show that the important 1A mutations happen soon after the first, and that therefore all important 1A mutations have roughly the same number of passengers, consider two successful mutations at times *s*_0_ and *s*_1_ which have sizes *W*_0_*e*^λ_1_(*t*−*s*_0_)^ and *W*_1_*e*^λ_1_(*t*−*s*_1_)^. For the second mutation to be larger, we’d need *W*_0_/*W*_1_ ≤ *e*^λ_1_(*s*_0_−*s*_1_)^. Since the cdf of the quotient of two exponentials with the same rate is *P*(*W*_0_/*W*_1_ ≤ *x*) = *x*/(*x*+ 1), we find that

$$\begin{array}{c} P(W_{0}/W_{1}\leq e^{\lambda_{1}\left(s_{0}-s_{1}\right)})=\frac{1}{e^{\lambda_{1}\left(s_{1}-s_{0}\right)}+1}. \end{array}$$

If *s*_1_ = *s*_0_ + 4/λ_1_ = *s*_0_ + 200, then the probability that the second mutation is larger is (1 + *e*^4^)^−1^ = 0.018. Thus, in our concrete example the most significant mutants occur within 200 time units of the first successful mutation. The mean number of mutations in 200 units of time is 200*ν*.
